# False Negative NIPT Results: Risk Figures for Chromosomes 13, 18 and 21 Based on Chorionic Villi Results in 5967 Cases and Literature Review

**DOI:** 10.1371/journal.pone.0146794

**Published:** 2016-01-15

**Authors:** Diane Van Opstal, Malgorzata I. Srebniak, Joke Polak, Femke de Vries, Lutgarde C. P. Govaerts, Marieke Joosten, Attie T. J. I. Go, Maarten F. C. M. Knapen, Cardi van den Berg, Karin E. M. Diderich, Robert-Jan H. Galjaard

**Affiliations:** 1 Department of Clinical Genetics, Erasmus Medical Center, Rotterdam, the Netherlands; 2 Department of obstetrics and prenatal medicine, Erasmus Medical Center, Rotterdam, the Netherlands; 3 Stichting Prenatale Screening Zuidwest Nederland, Wytemaweg 80, 3015 CN, Rotterdam, the Netherlands; VU University Medical Center, NETHERLANDS

## Abstract

Non-invasive prenatal testing (NIPT) demonstrated a small chance for a false negative result. Since the “fetal” DNA in maternal blood originates from the cytotrophoblast of chorionic villi (CV), some false negative results will have a biological origin. Based on our experience with cytogenetic studies of CV, we tried to estimate this risk. 5967 CV samples of pregnancies at high risk for common aneuplodies were cytogenetically investigated in our centre between January 2000 and December 2011. All cases of fetal trisomy 13, 18 and 21 were retrospectively studied for the presence of a normal karyotype or mosaicism < 30% in short-term cultured (STC-) villi. 404 cases of trisomies 13, 18 and 21 were found amongst 5967 samples (6,8%). Of these 404 cases, 14 (3,7%) had a normal or low mosaic karyotype in STC-villi and therefore would potentially be missed with NIPT. It involved 2% (5/242) of all trisomy 21 cases and 7.3% (9/123) of all trisomy 18 cases. In 1:426 (14/5967) NIPT samples of patients at high risk for common aneuploidies, a trisomy 18 or 21 will potentially be missed due to the biological phenomenon of absence of the chromosome aberration in the cytotrophoblast.

## Introduction

The validation of non-invasive prenatal testing (NIPT) for fetal trisomy detection revealed that there is a small chance of a false positive and false negative result [1]. Although technical limitations may explain these false results, both also have a biological basis. The fact that cell free ‘fetal’ DNA in the maternal plasma fraction originates from the cytotrophoblast of chorionic villi (CV) explains at least a part of the discrepancies between NIPT results and the actual fetal karyotype [2–4].

**Fig 1:**
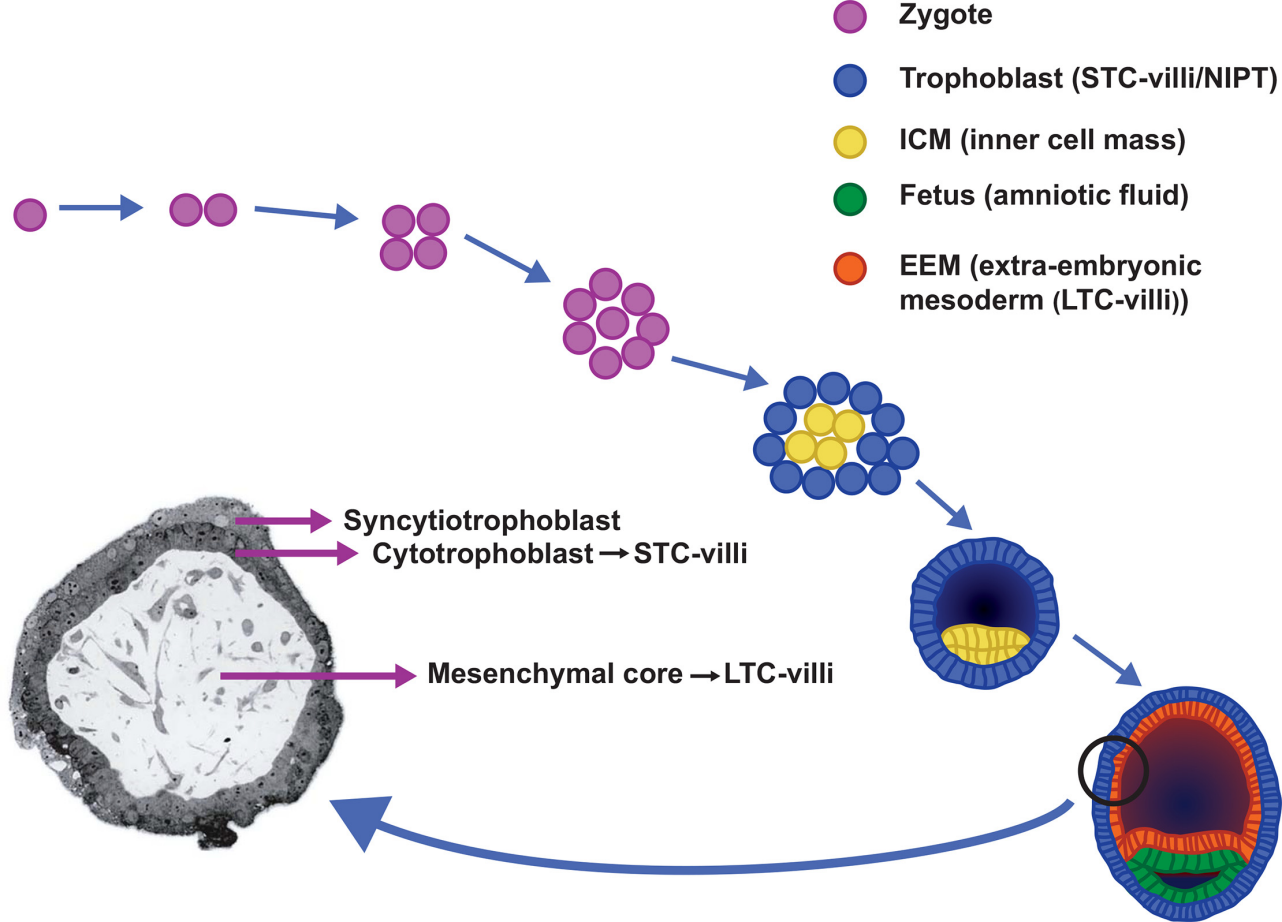
Early embryonic development from zygote to blastocyst. The cytotrophoblast which is studied in short-term cultured villi (STC-villi) and with NIPT is derived from the trophoblast of the blastocyst, whereas the mesenchymal core, investigated in long-term cultured villi (LTC-villi) originates from the extra-embryonic mesoderm (EEM). Both EEM and fetus are derived from the inner cell mass (ICM) of the blastocyst.

In order to understand the biological origin of false positive and false negative NIPT results, it is important to understand the cytogenetics of chorionic villi. CV can be processed for cytogenetic studies in two ways: the direct or semi-direct technique [5, 6], also called short-term cultured villi (STC-villi) and the long-term preparation method (long-term cultured villi (LTC-villi)) [7]. The origin of the cells that are investigated in the cytogenetic preparations are essentially different in both techniques: cells in STC-villi are derived from the cytotrophoblast, the outer cell layer of CV, and those of LTC-villi are predominantly from the inner cell layer, the mesenchymal core (Fig 1). The gold standard for cytogenetic analysis of CV is investigation of both STC (cytotrophoblast)- and LTC (mesenchymal core)-villi [8]. With NIPT, only DNA from the cytotrophoblast is investigated and therefore the results will be comparable to those from STC-villi.

As early as in the Eighties chromosome analyses of STC- and LTC-villi, have revealed that the chromosomal constitution of the cytotrophoblast may differ from that of the mesenchymal core and that of the fetus [9]. This is the consequence of the different embryonic origins of the three compartments mentioned. The cytotrophoblast is derived from the trophoblast of the blastocyst, whereas the mesenchymal core of the CV as well as the fetus itself are derived from the inner cell mass (ICM) (Fig 1). More particularly, the mesenchymal core originates from the hypoblast of the ICM and the fetus itself from the epiblast [10, 11]. Postzygotic mitotic division errors in chromosomally normal as well as abnormal conceptuses will lead to chromosomal mosaicism which is found in about 1 to 2% of CV [12]. Due to the uneven distribution of abnormal cells over the different compartments, the karyotypes of cytotrophoblast, mesenchymal core and fetus may be different [13]. In most of these mosaic cases the chromosomally abnormal cell line is confined to the cytotrophoblast and/or the mesenchymal core of CV, while the fetus itself has a normal karyotype [8, 12]. This phenomenon is called confined placental mosaicism (CPM) [14] and it accounts for 72% of all cases of mosaicism detected in CV [15]. Of the three possible types of CPM, the one with a (mosaic) chromosome aberration confined to the cytotrophoblast and not present in mesenchymal core or fetus, namely CPM type 1, is the most prevalent. CPM type 1 will cause a false positive NIPT result if the percentage of abnormal cells in the cytotrophoblast is sufficiently high [16]. The same is true for the less prevalent CPM type 3 with chromosomally abnormal cells restricted to both cell layers of the CV and absent in the fetus itself.

On the other hand, chromosomal mosaicism may also lead to false negative NIPT results. Two types of mosaicism are associated with a normal karyotype in the cytotrophoblast while the fetus itself has a chromosome aberration. Firstly, generalised mosaicism confined direct normality (GMDD)[12], characterized by the presence of a chromosome aberration in the fetus and mesenchymal core of the placenta, with the cytotrophoblast being chromosomally normal. Secondly, although extremely rare, confined fetal mosaicism (CFM) with a normal karyotype in STC-and LTC-villi and with ultimately abnormal cytogenetic results in the fetus [12, 17]. Both types of mosaicism will show normal NIPT results due to a normal karyotype in the cytotrophoblast, the fetus having an abnormal karyotype though. The types of generalized mosaicism with absolute or relative concordance (GMAC or GMRC, respectively) may potentially also go undetected with NIPT if the percentage of abnormal cells in STC-villi is below a certain threshold [18].

In clinical practice, it is now generally accepted that if NIPT reveals a trisomy 13, 18 or 21, follow-up investigations by an invasive procedure, preferentially amniocentesis, are necessary to confirm the results, because NIPT is not fully diagnostic [19–21]. This will reveal false positive results and by doing so, an unnecessary termination of pregnancy may be prevented. However, it is as important to realize that if the NIPT result is normal, there is a possibility that this result is falsely negative.

Little information is available with regard to false negative NIPT results, apart from a few reported cases [1]. Studies on false negative NIPT results would require thorough follow-up investigations of all tested pregnancies, including cytogenetic investigations of those resulting in a miscarriage or intrauterine fetal demise. In order to study the risk for a biological false negative NIPT result involving the chromosomes 13, 18 and 21, we retrospectively investigated all CV cases of fetal trisomy 13, 18 and 21 that were found in our centre during a 12-year period (January 2000-December 2011). We determined the proportion of cases with normal or low-level mosaicism in STC-villi that potentially would lead to false negative NIPT results.

## Materials and Methods

All cases of fetal trisomy 13, 18 and 21 among 5967 CV samples that were cytogenetically investigated in our centre during a 12-year period (January 2000-December 2011) were retrospectively studied for the presence of a normal karyotype or low-level mosaicism < 30% in STC-villi. The samples were obtained under continuous ultrasound guidance by transabdominal aspiration. The threshold of 30% was chosen based on previously published papers [16, 22]. The main indications for CV sampling in these trisomic cases were fetal ultrasound abnormalities, increased nuchal translucency, advanced maternal age > 36 years, and/or abnormal first trimester screening results (risk >1:200). Patients undergoing prenatal diagnosis at our medical university are informed that we may investigate (publish) their medical data as long as all data remain anonymised. Each patient had the opportunity to object to this. No objections were made.

This study overlaps with the study of van den Berg et al. (2006) which describes the results in 2389 CV samples investigated during the time period November 2000 and July 2005.

Despite our standard protocol during the study period in which we routinely performed STC- as well as LTC-villi, in most trisomy 21 cases only STC villi were analysed since this chromosome aberration can be considered a “certain abnormality” when encountered in STC-villi, irrespective of the indication[8]. Similarly, in most cases of a fulblown trisomy 13 and 18, the karyotyping was restricted to that of STC-villi if fetal ultrasound anomalies, detected at the time of CV sampling matched the chromosome aberration.[8]. In all other cases (normal or mosaic results in STC-villi or 100% trisomy 13 or 18 in STC-villi without ultrasound abnormalities), LTC-villi were investigated as well. GTG banding was routinely used in all cases. At least 8 metaphases in STC-villi (range 8–21) and 10 in LTC-villi (range 10–25) were studied. Additionally, in most mosaic cases FISH on interphase nuclei was performed in order to exclude the presence of low-level mosaicism in karyotypically normal STC-villi, or in order to further study the level of mosaicism in mosaic STC and/or LTC-cases.

## Results

On 5967 CV samples, a total of 404 (6.7%) fetal trisomies 13, 18 and 21 were found. All these cases were considered to be true cases of fetal trisomy due to:

1. the presence of 100% trisomy in LTC-villi
2. the presence of 100% trisomy 13 or 18 in STC-villi in association with fetal ultrasound abnormalities that matched the abnormal karyotype
3. the presence of 100% trisomy 21 in STC-villi, irrespective of the indication as described by van den Berg et al. [8, 23].

Of these 404 cases, 14 (3.7%) had a normal (N = 9) or low-mosaic (<30%) (N = 5) karyotype in STC-villi and therefore would potentially be missed if NIPT was performed in these cases (Table 1). This means that in 14/5967 high risk pregnancies a trisomy will potentially be missed with NIPT based on biological grounds.

**Table 1:** Number of fetal trisomy 13, 18 and 21 cases amongst 5967 CV samples with karyotypically normal results or low-level mosaicism <30% in STC-villi.

Chromosome aberration	Total number of trisomic cases	Number with normal STC	Number with mosaic STC<30%	Total number of normal or mosaic < 30% STC (%)
Trisomy 21	242	3	2	5 (2,0%)
Trisomy 18	123	6	3	9 (7.3%)
Trisomy 13	39	0	0	0
Total	404	9	5	14 (3,5%)

**Trisomy 21** (Table 2): 242 cases of fetal trisomy 21 were encountered among 5967 CV samples of which 5 (2.0%) showed karyotypically normal STC results (N = 3) or low-level mosaicism (N = 2). In two out of three cases with karyotypically normal STC, additional FISH on 200 nuclei revealed low-level mosaicism of 23% and 8%. LTC-villi in these cases showed a non-mosaic trisomy 21 karyotype. The indications for sampling were an increased nuchal translucency (N = 3), ultrasound anomalies (N = 1) and abnormal first trimester screening result without an enlarged nuchal translucency (N = 1). Nine cases of high-level mosaicism (> 30%) in STC-villi associated with 100% trisomy 21 in LTC-villi were found. Those cases were assumed to be detectable with NIPT.

**Table 2:** Details of trisomy 21 cases with normal or low-mosaic (<30%) results in STC-villi in our cohort of 5967 CV samples.

	Indication	Karyotype STC-villi (% +21)	FISH STC: % +21 (number of cells)	Karyotype LTC-villi	FISH LTC: % +21 (number of cells)	Confirmatory studies
1	Hygroma colli/ AMA37	46,XY[10] (0%)	23% interphase nuclei (N = 200)	47,XY,+21[11]	----	---
2	NT 6mm/ AMA36	46,XX[8] (0%)	0% metaphases (N = 38)	47,XX,+21[16]	100% metaphases (N = 44)	Skin biopsy: 100% interphase nuclei (N = 50)
3	ftCT 1:10	46,XY[11] (0%)	8% interphase nuclei (N = 205)	47,XY,+21[21]	100% interphase nuclei (N = 100)	---
4	NT 6.9mm/ ftCT 1:2	47,XY,+21[4]/46,XY[11] (27%)	---	47,XY,+21[19]	---	---
5	NT 5.5mm/ ftCT 1:5	47,XX,+21[3]/46,XX[17] (15%)	---	47,XX,+21[16]	---	---

AMA: advanced maternal age (≥ 36yrs); NT: nuchal translucency; ftCT: abnormal first trimester combined screening test results; STC-villi: short-term cultured villi; LTC-villi: long-term cultured villi

**Trisomy 18** (Table 3): a total of 123 cases of fetal trisomy 18 were found in 5967 CV samples of which 9 (7.3%) showed normal (N = 6) or low mosaic abnormal (N = 3) STC results and would likely be missed with NIPT. Interphase FISH (100–200 nuclei) excluded mosaicism in the six normal cases. The indication for CV sampling was the detection of fetal ultrasound abnormalities in all cases. One case of high level mosaicism (87%) in STC-villi was found and assumed to be detectable with NIPT.

**Table 3:** Details of trisomy 18 cases with normal or low-mosaic (<30%) results in STC-villi in our cohort of 5967 CV samples.

	Indication	Karyotype STC-villi (% +18)	FISH STC % +18 (N≥100 nuclei)	Karyotype LTC-villi	FISH LTC % +18 (N≥100 nuclei)	Confirmatory studies
1	US: omphalocele, hygroma colli, hydrops foetalis; hydrothorax	46,XX[8] (0%)	0%	47,XX,+18[14]/46,XX[2]	~100%*	Skin: 100% metaphases (N = 61),and ~100%* interphase nuclei (N = 100)
2	US: IUGR	46,XX[10] (0%)	0%	47,XX,+18[16]	~100%*	Skin: ~100%* interphase nuclei (N = 100)
3	US: hydrops foetalis, cor vitium, abdominal wall defect	46,XX[9] (0%)	0%	47,XX,+18[25]	---	---
4	US: NT 8 mm, IUGR	47,XY,+18[3]/46,XY[18] (14%)	0%	47,XY,+18[9]/46,XY[1]	~100%*	---
5	US: omphalocele, NT 6 mm, ftCT 1:2	46,XX[10] (0%)	0%	47,XX,+18[9]	~100%*	---
6	US: hydrops foetalis, IUD	46,XY[7] (0%)	0%	47,XY,+18[7]/46,XX[7] (MCC)	~100%* (in Y-positive nuclei)	---
7	US: hydrops foetalis, ascites, NT 7 mm	46,XY[9] (0%)	0%	47,XY,+18[8]/47,XY,+2[10]	38% +18 (and 32% +2)	AF: ~100%* interphase nuclei (N = 165)
8	US: encephalocele, IUGR	48,XY,+mar,+18[1]/47,XY,+18[2]/47,XY,+mar[11]/46,XY[5] (16%)	FISH for mar identification: mar = der(18)(L1.84+, WCP18-)	47,XY,+18[11]	~100% *	Skin: ~100%*
9	US: hygroma colli	47,XX,+18[2]/46,XX[18] (10%)	0%	47,XX,+18[16]	---	---

US: ultrasound abnormalities; IUGR: intrauterine growth restriction; IUD: intrauterine death; NT: nuchal translucency; ftCT: abnormal first trimester screening results; AF: uncultured amniotic fluid cells; MCC: maternal cell contamination; STC-villi: short-term cultured villi; LTC-villi: long-term cultured villi

* The % of nuclei with 3 signals with probe L1.84 (18 centromere probe) varied between 70 and 100%, fitting a non-mosaic trisomy 18 according to our protocol.

**Trisomy 13**: a total of 39 cases of trisomy 13 were found and in all cases a 100% (n = 38) or high mosaic (86%) karyotype was found in STC-villi. Therefore, none of the trisomy 13 cases would potentially be missed with NIPT. The indication for CV sampling was the detection of fetal ultrasound abnormalities in all cases.

## Discussion

Cell free ‘fetal’ DNA in maternal blood originates from the cytotrophoblast [2] and is not always concordant with true fetal DNA. This will explain at least some of the false positive and false negative NIPT results that are currently published. After the introduction of chorionic villus (CV) sampling as an alternative to amniocentesis in the early Eighties, for a short time, cytogenetic studies in chorionic villi were performed solely on preparations from the cytotrophoblast in many laboratories around the world [5]. However, soon after its introduction we learned that the karyotype of this cell layer, which is derived from the trophoblast of the blastocyst [10] is not always representative for that of the fetus. This is due to chromosomal mosaicism with uneven distribution of normal and abnormal cells over the different compartments (trophoblast, extraembryonic mesoderm and fetus) of a conceptus [12, 24]. This phenomenon led the publication of many papers on false positive and negative results in CV and to a change of protocol advocating the use of two culture methods: STC-villi investigating the cytotrophoblast and LTC-villi investigating the mesenchymal core of CV. It was shown that only the analysis of both methods will lead to a high degree of accuracy [9]. With the introduction of NIPT “l’histore se repète”. Moreover, due to the origin of the cell free ‘fetal’ DNA and the possibility of chromosomal mosaicism, a 100% sensitivity and specificity for NIPT can and will never be reached.

Knowing that with NIPT we are investigating the cytotrophoblast of CV, risk figures for false positive and false negative NIPT results should be derivable from CV figures. Therefore, we performed a retrospective analysis of our CV data of 5967 patients at high risk for the commona aneuploidies, mainly AMA > 36 years, ftCT (abnormal first trimester combined test results > 1:200), increased NT and other ultrasound abnormalities. Cases of chromosomal mosaicism with normal results or low level mosaicism < 30% in STC-villi and a full blown trisomy 13, 18, 21 in LTC-villi and/or fetus were collected in order to determine the potential incidence of false negative NIPT results. The present study shows that in 0.2% (14/5967) (95% CI 0.13%-0.39%) of patients at high risk for common aneuploidies, a false negative NIPT result for trisomy 13, 18 and 21 can be expected. This figure seems to be higher than the figures on false negative STC-villi reported in the literature (see Table 4). This is partially explained by the inclusion of low mosaic cases in our presented data, so that for a sound comparison these mosaic cases preferably should be excluded. Doing this, the false negative rate in our series in fact would still be 0.15% (9/5967) (95% CI 0.07%-0.29%)(Table 1), which is in agreement with our previously published data (van den Berg et al. 2006), but would still be higher than the few published figures (see Table 4). The most likely explanation is the difference in indications for sampling shown by a lower incidence of trisomies 13, 18 and 21 in the paper of Grati et al. [25] (3% versus 7% (404/5967) in our patient group) (Table 4). This is also confirmed by the paper of Toutain et al. [26, 27] who found an incidence of GMDD of 1/386 (0.26%) in a small series of CV sampled for increased nuchal translucency or other ultrasound abnormalities. So, depending on whether there is a high or low risk for aneuploidy, the estimated chance for a false negative NIPT diagnosis will vary between 0.02% and 0.26% (Table 4). However, a few things should be taken into account. Firstly, these figures are probably overestimated due to the well-known placental variation in cases of mosaicism. Therefore, it cannot be excluded that a CV biopsy in the first trimester may miss cytogenetically abnormal cotylydones that probably will lead to abnormal NIPT results assuming deposition of the whole placental cytotrophoblast into the maternal circulation. However, in our opinion we consider the risk to be minimal since all samples were obtained under continuous ultrasound guidance by transabdominal instead of transcervical aspiration. Secondly, based on current experience [16, 22] a mosaic of less than 30% is expected to be missed with NIPT. However, this for sure will highly depend on fetal fraction with a 100% trisomy potentially missed if fetal fraction is too low and a ≤ 30% mosaic detected if fetal fraction is high.

**Table 4:** False negative cytogenetic results in STC-villi involving trisomy 13, 18 and 21: literature review.

Reference	Total number of CV	Number of false negative STC (%)	Number of trisomies 13, 18, 21 on total number of samples (%)
Grati et al. 2014	52,673	15 (0.03%)	1599 (3.0%)
Battaglia et al. 2014	7112	3 (0.04%)	*Not given*
Smith et al. 1999	7934	7 (0.09%)	*Not given*
Pittalis et al. 1994	4860	1 (0.02%)	87 (1.8%)
Van den Berg et al. 2006	2389	4 (0.17%)	*Not given*
Toutain et al. 2010	386	1 (0.26%)	70 (18.1%)
Present study	5967	9 (0.15%)	404 (6.7%)

Especially the risk for a false negative trisomy 18 is obvious, with in the present study 7.3% of all fetal cases with a trisomy 18 potentially being missed if NIPT was performed. The preferential involvement of trisomy 18 in GMDD can also be recognised in the recent paper of Grati et al. [25] (6 out of 382 cases (1,6%)), but also in the former CV literature (ACC CVS database 2005). In fact, Porreco et al. [28] who conducted a prospective multicenter observational study comparing NIPT with the results of invasive testing in 3430 cases at high risk of common aneuploidies, found 3 out of 38 cases of trisomy 18 (7.9%) to have a false-negative NIPT result.

It is reasonable to think that the actual prevalence of false negative NIPT results may even be higher than the figures mentioned. False negative results due to technical problems such as a low fetal DNA fraction for instance as a consequence of high BMI [29, 30, 31] or of some fetal aneuploidies [32] are a well know problem and should be added to these figures. On the other hand, the risk of a false negative NIPT result due to confined fetal mosaicism (CFM) can probably be ignored. This type of mosaicism is extremely rare, probably less than 0.003%, although absolute figures are not available due to incomplete follow-up investigations [17].

So far little information is available on the true prevalence of false negative NIPT results. An emerging number of case reports on this topic are published though [1]. Therefore, based on the estimated risk of missing 3.5% (14/404) of fetal trisomies 13, 18 and 21 with NIPT, as shown in this paper, we would like to recommend follow-up cytogenetic testing in all cases with normal NIPT results if fetal anomalies are detected by ultrasound, if a miscarriage or intrauterine death occurs and if a syndromally abnormal child is born. In such cases we would like to encourage cytogenetic testing of both fetus or child and placenta. This will ultimately lead to a better knowledge on the true prevalence of false negative NIPT results.

In conclusion, a retrospective analysis of ~6000 CV samples of patients at high risk of aneuploidy revealed that in 1:426 (14/5967) pregnancies a fetal trisomy 13, 18, 21 may potentially be missed with NIPT due to absence of the chromosome aberration in the cytotrophoblast. Moreover, irrespective of the indication we could calculate that 2% (5/242) and 7.3% (9/123) of trisomies 21 and 18, respectively, may likely not be detected with NIPT due to a chromosomally normal or low mosaic cytotrophoblast. Due to the relatively small number of cases, for trisomy 13 this figure remains unclear. Since most trisomic fetuses, especially those with trisomy 13 or 18, will show anomalies that are detectable by ultrasound, part of these false negatives will be discovered later on during pregnancy or will end in an intrauterine death. It is important that patients opting for NIPT are informed about both the technical and biological limitations of the non-invasive prenatal procedure so that an informed choice can be made between non-invasive targeted testing and invasive sampling with cytogenetic testing of CV or amniocytes [33]. In the meanwhile, thorough follow-up investigations in cases of normal NIPT results will ultimately lead to the real figures for NIPT sensitivity.
